# Associations Between Children’s Physical Activity, Pain and Injuries

**DOI:** 10.1177/00315125211028455

**Published:** 2021-06-29

**Authors:** João Paulo de Aguiar Greca, Thomas Korff, Jennifer Ryan

**Affiliations:** 1 College of Health, Medicine and Life Sciences, Brunel University London, UK; 2 Population Health Sciences Institute, Faculty of Medical Sciences, Newcastle University, Newcastle upon Tyne, UK; 3 Frog Bikes, Ascot, UK; 4 Department of Public Health and Epidemiology, RCSI University of Medicine and Health Sciences, Dublin, Ireland

**Keywords:** exercise behaviour, fitness, exercise and sport, physical stressors, child motor development

## Abstract

Our aim in this study was to investigate the relationships between physical activity (PA), pain, and injury among children. Secondarily, we examined whether these relationships differed between children with normal versus excessive weight or obesity. This was a cross-sectional study of 102 children (57 girls) aged 8–12 years old. We assessed the prevalence of moderate and vigorous PA using accelerometry over a seven-day period. We examined the associations between moderate PA, vigorous PA, pain presence, and injury presence using generalized estimating equations with a logit link and binomial distribution. We adjusted the obtained models for potential confounders and explored the moderating effect of weight status. We found no association between moderate PA and pain, but time spent in vigorous PA was associated with pain. Neither moderate or vigorous PA were associated with injury, and there was no moderating effect of weight status in these relationships. In summary, we found that objectively measured vigorous PA is associated with pain among 8–12 year old children. While these results should be replicated in longitudinal studies, they suggest that an association between vigorous PA and pain should be considered when developing PA interventions for children.

## Introduction

Physical activity (PA) is a broad concept, defined as any bodily movement generated by skeletal muscle that results in energy expenditure (Caspersen et al., 1985). According to the UK Chief Medical Officers' Physical Activity Guidelines (Davies et al., 2019), children should engage in moderate-to-vigorous physical activity (MVPA) for an average of at least 60 minutes daily across the week. PA can be accumulated in short bouts when performed as part of the child’s daily routine (Davies et al., 2019). However, many children do not achieve these PA recommendations (Hallal et al., 2012), and pain and injuries may act as a barrier to PA participation for some children (Smith et al., 2014). In particular, weight-bearing PA, such as habitual walking and running, may result in pain and injury because of increased joint loading (Greca et al., 2019). Yet, only a few studies have examined any association between PA and pain and injuries among children. Understanding the association between PA, pain and injuries in children may support the need to develop interventions to increase PA in this population (H. Wilkie et al., 2016).

Silva et al. (2017) found that self-reported time in moderate intensity PA (MPA) was associated with a higher probability of participants reporting neck, shoulder, low back, wrist, hip, knee and ankle/foot pain. These authors also found that time in vigorous intensity PA (VPA) was associated with a higher probability of reporting shoulder, mid back, knee and ankle/foot pain. Conversely, Swain et al. (2016) found that girls who reported reduced participation in MVPA were more likely to have back pain, headache and stomach-ache and that boys who reported reduced participation in MVPA were more likely to have stomach-aches or headaches. A third study found that self-reported frequent participation in MPA was associated with higher odds of injury among boys, while frequent participation in VPA was associated with higher odds of injury among girls (Lowry et al., 2007).

While the aforementioned studies suggest that PA may be positively associated with pain or injuries, these investigators used subjective self-report measures of PA that are likely to have been inaccurate measures of PA duration and intensity (Hidding et al., 2018). Only one study has examined the association between objectively measured PA and pain in children (Aartun et al., 2016), and these authors found no association between different levels of PA (i.e. low, moderate and vigorous) and neck or back pain from either cross-sectional or longitudinal analyses. However, these authors focused only on neck and back pain and did not look at any separate association between MPA and pain versus VPA and pain. Nauta et al. (2017) found an association between objectively assessed MVPA and injuries, but these investigators assessed only upper extremity injuries.

From this review, it is apparent that there has been insufficient research investigating associations between objectively measured MPA or VPA and pain or injuries in children and no research addressing whether children who are overweight (OW) or obese (OB) may be particularly likely to develop pain and injury due to excess joint loading during weight-bearing activities (Paulis et al., 2014). Thus, in the present study, we aimed to improve upon earlier research in these ways.

## Method

### Study Design and Participants

This study used a cross-sectional research design. Data collection took place in London, England and occurred from December of 2016 until March of 2017. We studied a convenience sample of 8–12 year old children who were from three schools in the London Borough of Hillingdon over 18 months, starting in 2015. These three schools enrolled approximately 1,095 8–12-year-old students. Head teachers sent participant information sheets and consent forms to parents of potentially eligible children, and participating children returned consent forms signed by their parents and provided their own written assent. We received ethical approval for this research protocol from the Department of Life Sciences Research Ethics Committee at Brunel University London (reference number 2440-MHR-Mar/2016-2773-2). We excluded children from participation if they had a disability or medical condition that prevented them from engaging in daily PA as assessed by the *Physical Activity Readiness Questionnaire* (PAR-Q) (Arraiz et al., 1992; Shephard, 2015; Thomas et al., 1992).

### Procedure

Over a two-day period, a single researcher collected data on site at the schools where participants were enrolled. On the first day, participants could ask questions about the study and decline to participate, before accelerometers, PA diaries and instruction sheets about accelerometer usage were distributed. After seven days, the accelerometers and the PA diaries were collected, anthropometric measurements were obtained, and questionnaires regarding pain, injury and socioeconomic status were completed by participants.

### Body Composition

We measured the children’s stature to the nearest 0.1 cm using a calibrated stadiometer (Charder HM200P PortStad Stadiometer) and assessed their body weight to the nearest 0.1 kg using a calibrated electronic weight scale (Seca, Hamburg, Germany). We calculated their body mass index (BMI) as weight (in kg) divided by stature (in m) squared. Children were categorized as normal weight or OW/OB using the extended international (International Obesity Task Force) BMI cut-offs for thinness, OW and OB (Cole & Lobstein, 2012). We measured and collected waist circumference data using a Gulick anthropometric tape (Creative Health Products, Plymouth, USA), recording waist and hip circumferences to the nearest 0.1 cm and measuring circumference horizontally at the midpoint between the inferior border of the bottom rib and the top end of the iliac crest. We assessed body fat using skinfold measurement of the triceps and medial calf sites collected on the right side of the body using a Harpenden Skinfold Caliper (Country Technologies). We estimated participants’ body fat by the relative body fat for girls and boys using specific equations proposed by Slaughter et al. (1988).

### Socioeconomic Status

We assessed participants’ socioeconomic status using the Family Affluence Scale (Currie et al., 2008). The Family Affluence Scale is a questionnaire developed specifically for young students, and it aims to reflect a family’s money expenditures (Currie et al., 1997). The questionnaire was updated in 2008 and was found to be valid as well as reliable and suitable for students (Currie et al., 2008). Essentially, the Family Affluence Scale explores socioeconomic inequalities by classifying a set of items that reflects a family’s assets and consumption. The questionnaire considers items such as the number of cars that a family possesses, whether a child occupies their own bedroom, the number of times that the family went on holidays during the past 12 months and the number of computers the family has. This questionnaire has been widely used in studies of children (Frasquilho et al., 2017; Voráčová et al., 2016) and in research exploring the occurrence of injuries and PA (Pickett et al., 2005; Warsh et al., 2010). A score from zero to nine permits categorization into tertiles representing low (0 to 3), middle (4 to 6) and high (7 to 9) affluence groups (Currie et al., 2008).

### Injury

Participants were asked to report injury with the following question: “During the past 12 months, how many times were you injured and had to be treated by a doctor or nurse?” Injury in children has been widely investigated using this question (Addor & Santos-Eggimann, 1996; Pickett et al., 2005; Warsh et al., 2010).

### Pain

Pediatric pain has been previously described as a subjective issue and is commonly reported by healthy children (Anthony & Schanberg, 2003). In this study, participants were asked to self-report any pain or discomfort, of the whole body, that they experienced over the seven days of testing using a visual analog scale from the validated and reliable Pediatric Pain Questionnaire (Gragg et al., 1996; Varni et al., 1987). With this method participants mark a point on a 100 mm horizontal line anchored by several faces representing “no pain” to “severe pain” (Cohen et al., 2008). Visual analog scales have been widely recommended as the most appropriate and valid method for assessing children’s pain (Huguet et al., 2010; Rapoff, 2003; Stinson et al., 2006).

### Habitual Physical Activity

We assessed PA objectively with a triaxial ActiGraph wGT3X-BT accelerometer (Pensacola, USA). The ActiGraph wGT3X-BT monitor is a small (4.6 cm×3.3 cm x 1.5 cm) and light-weight device (19 g) with which we used a 15-second epoch and sampling rate of 30 Hz. Participants were requested to wear the accelerometer around their waist at the right hip (Trost et al., 2005) for all waking hours except when swimming, showering or during other water activities. Participants were asked to wear the device for seven days (Trost et al., 2005).

We used the Actilife 6® software to download and process all data recorded. We established an upper limit of 20,000 counts per minute as a threshold to avoid spurious data or monitor failure (Haapala et al., 2017; Heil et al., 2012). Non-wear time was defined as 60 minutes or more of consecutive zero counts (Aartun et al., 2016; Toftager et al., 2013). Evidence has shown that, when using accelerometry, two to three days of monitoring PA are required to attain a reliability coefficient of 0.70 in primary school children (Trost et al., 2000). Thus, an average of at least three days was used to report children’s PA. Minimum wear time per day was established as at least 500 minutes of recorded PA per day (Haapala et al., 2017; Hinkley et al., 2012). Cut-points were 3581 to 6129 counts per minute for MPA and ≥6130 counts per minute for VPA, which have been validated among children (Mattocks et al., 2007).

### Statistical Analysis

We assessed data distributions of variables of interest using Q–Q (quantile-quantile) plots and histograms. Data distributions that were normally distributed were described using means and standard deviations. Variables with skewed distributions were described using medians and interquartile ranges. Categorical variables were presented as frequencies and percentages. We compared the odds of experiencing pain over the past seven days between children with and without OW/OB using generalized estimating equations with a logit link, binomial distribution and an exchangeable correlation matrix to account for clustering within schools. We used the same method to compare the odds of experiencing an injury between children with and without obesity. We compared moderate and vigorous physical activity between children with and without OW/OB using generalized estimating equations with an identity link and Gaussian distribution.

We examined associations between MPA, VPA and presence of pain, and associations between MPA, VPA and injury using generalized estimating equations with a logit link and binomial distribution. We first fitted unadjusted models before adjusting for potential confounders. When examining the association between PA and pain, we adjusted for age, sex, socioeconomic status, waist circumference, BMI category and presence of injuries over the past 12 months. When examining the association between PA and injuries, we adjusted for age, sex, socioeconomic status, waist circumference, and BMI category.

We additionally included MPA-by-BMI and VPA-by-BMI interaction terms, respectively, in adjusted models to examine if the association between PA and pain and PA and injuries differed according to weight status. Statistical analyses were performed using STATA (StataCorp LLC, College Station, Texas, USA), version 13. Statistical significance for all analyses was set a *p* < .05.

## Results

One hundred fourteen children participated in the study. No child was excluded from the study based on their response to the PAR-Q, but 12 students were excluded from the final analyses for at least three days of missing PA data, leaving 102 children in the final analyses. Participant characteristics are described in Table 1. Mean participant age was 10.4 (*SD* = 1.2) years and 52% of participants were female. Most participants (65%) had normal weight. Participants wore the accelerometer for a median of five days (minimum = 3 days and maximum = 7 days). Forty percent reported pain in the past seven days, and 28% reported an injury in the past 12 months. There was no difference in the presence of pain (odds ratio [OR]: 0.77, 95% CI 0.35 to 1.68, *p* = .513) or of injury (OR: 1.19, 95% CI 0.31 to 4.57, *p* = .798) between children with normal weight and those with OW/OB. Participants engaged in MPA for a mean of 88.8 (*SD* = 51.9) minutes per day and in VPA for a mean of 17.1 (*SD* = 20.4) minutes per day. There was a difference in MPA (β: 3.1, 95% CI −0.3 to 6.4, *p* = .071) between children with normal weight and those with OW/OB, but there was no difference in VPA (β: 0.5, 95% CI −5.3 to 6.3, *p* = .859) between these weight-based groups.

**Table 1.** Participant Characteristics.

	Normal weight n = 66		OW/OB n = 36		Total n = 102	
	n (%)	Mean (*SD*)	n (%)	Mean (*SD*)	n (%)	Mean (*SD*)
Age, yr		10.4 (1.2)		10.4 (1.1)		10.4 (1.2)
Female, n (%)	31 (47.0)		22 (61.1)		53 (52.0)	
Body mass, kg		34.3 (8.0)		49.2 (10.5)		39.5 (11.4)
Stature, cm		142.0 (10.4)		147.2 (12.0)		143.8 (11.2)
BMI, kg/m^2^		16.8 (2.2)		22.4 (2.0)		18.8 (3.4)
Waist circumference, cm		53.8 (16.6)		60.8 (20.7)		56.2 (18.4)
Body fat (%)		21.9 (5.9)		33.2 (8.8)		25.9 (8.9)
Socioeconomic status, n (%)						
Low	8 (12.1)		5 (13.9)		13 (12.8)	
Middle	37 (56.1)		12 (33.3)		49 (48.0)	
High	21 (31.8)		19 (52.8)		40 (39.2)	
Pain (over the past seven days)	28 (42.4)		13 (36.1)		41 (40.2)	
Injury (over the past 12 months)	18 (27.3)		11 (30.6)		29 (28.4)	
MPA (min/day)		87.8 (49.8)		90.5 (56.1)		88.8 (51.9)
VPA (min/day)		17.0 (22.1)		17.4 (17.3)		17.1 (20.4)

BMI: body mass index. OW/OB: overweight/obesity; *SD*: standard deviation.

### Association Between PA and Pain

Table 2 presents the results for associations between PA and pain. In unadjusted analyses there was no evidence that MPA or VPA was associated with pain. In the adjusted analysis, MPA was not associated with pain (adjusted 95% OR: 1.00, 0.99 to 1.01, *p* = .147), nor was there a significant difference in the relationship between MPA and pain of children with normal weight versus those with OW/OB based on the *p* value for the BMI-by-MPA interaction term (*p* = .246). However, when children of different weight status were examined separately, there was evidence that MPA was associated with pain in children with normal weight (adjusted OR: 1.01, 95% CI 1.00 to 1.02, *p* = .014) but not in those with OW/OB (adjusted OR: 1.00, 95% CI 0.99 to 1.01, *p* = .885).

**Table 2.** Unadjusted and Adjusted Associations Between Physical Activity and Pain in Children.

	Unadjusted OR (95% CI)	*p*	Adjusted OR (95% CI)	*p*
MPA	1.00 (0.99 to 1.01)	.218	1.00 (.099 to 1.01)	.147
Age			0.91 (0.41 to 1.99)	.805
Sex: male^a^			2.70 (2.17 to 3.35)	<.001
SES: middle^b^			10.23 (6.81 to 15.37)	<.001
SES: high^b^			7.26 (3.30 to 15.97)	<.001
OW/OB^c^			0.80 (0.55 to 1.16)	.238
Waist circumference			1.02 (1.01 to 1.03)	.004
Injury			1.15 (0.59 to 2.25)	.529
VPA	1.02 (1.00 to 1.04)	.064	1.02 (1.00 to 1.04)	.016
Age			0.90 (0.38 to 2.13)	.817
Sex: male^a^			2.98 (2.14 to 4.16)	<.001
SES: middle^b^			9.65 (6.89 to 13.53)	<.001
SES: high^b^			5.89 (3.62 to 9.60)	<.001
OW/OB^c^			0.83 (0.55 to 1.25)	.368
Waist circumference			1.02 (1.01 to 1.03)	<.001
Injury			1.12 (0.62 to 2.04)	.568

CI: confidence interval; MPA: moderate physical activity; OW/OB: overweight/obesity; SES: socioeconomic status; VPA: vigorous physical activity

^a^Reference female.

^b^Reference lower SES category.

^c^Reference normal weight.

In the adjusted analysis, VPA was associated with pain (adjusted OR 95% CI: 1.02, 1.00 to 1.04, *p* = .016), but there was no significant difference in the association between VPA and pain when comparing children with normal weight and those with OW/OB (BMI-by-VPA interaction term *p* = .157). In this analysis, even separating the weight status groups revealed no evidence of a significant association between VPA and pain in either children with normal weight (adjusted OR: 1.04, 95% CI 0.98 to 1.12, *p* = .203) or children with OW/OB (adjusted OR: 1.00, 95% CI 0.99 to 1.01, *p* = .769).

### Association Between PA and Injuries

Table 3 presents associations between PA and injuries for MPA and VPA, respectively. There was no evidence from either unadjusted or adjusted models that MPA (adjusted OR: 1.00, 95% CI 0.99 to 1.01, *p* = .935) or VPA (adjusted OR: 1.00, 95% CI 0.99 to 1.02, *p* = .396) were associated with children’s injuries. Similarly, there was no evidence that the association between MPA and injury or VPA and injury differed between children with normal weight and children with OW/OB (BMI-by-MPA interaction term *p* = .851 and BMI-by-VPA interaction term *p* = .234).

**Table 3.** Unadjusted and Adjusted Associations Between Physical Activity and Injuries in Children.

	Unadjusted OR (95% CI)	*p*	Adjusted OR (95% CI)	*p*
MPA	1.00 (0.99 to 1.01)	0.492	1.00 (0.99 to 1.01)	.935
Age			1.88 (1.39 to 2.54)	<.001
Sex: male^a^			0.99 (0.44 to 2.20)	.975
SES: middle^b^			1.28 (0.17 to 9.89)	.812
SES: high^b^			1.05 (0.11 to 10.43)	.964
OW/OB^c^			1.26 (0.25 to 6.49)	.780
Waist circumference			0.99 (0.97 to 1.01)	.231
VPA	1.01 (1.00 to 1.02)	0.200	1.00 (0.99 to 1.02)	.396
Age			1.86 (1.25 to 2.76)	.002
Sex: male^a^			1.01 (0.45 to 2.28)	.983
SES: middle^b^			1.25 (0.17 to 9.43)	.826
SES: high^b^			1.00 (0.11 to 9.42)	.999
OW/OB^c^			1.27 (0.23 to 7.09)	.784
Waist circumference			0.99 (0.97 to 1.01)	.276

CI: confidence interval; MPA: moderate physical activity; OW/OB: overweight/obesity; SES: socioeconomic status; VPA: vigorous physical activity

^a^Reference female.

^b^Reference lower SES category.

^c^Reference normal weight.

## Discussion

The purpose of this study was to investigate associations between PA, pain and injuries in 8–12-year-old children. We found that VPA was positively associated with pain, but MPA was only associated with pain among children with normal weight (and not among children with OW/OB). There was no evidence that MPA or VPA were associated with injury. There was no evidence of an association between MPA and injury or VPA and injury and no evidence that children with normal weight and children with OW/OB differed in the presence of any such association.

This is the first study investigating whether objectively measured moderate and vigorous PA are associated with children’s pain and injuries. Only one study has investigated the association between objectively measured PA intensity and pain, specifically back and neck pain, in children. Aartun et al. (2016) found no association between MVPA or VPA and spinal pain in a sample of 906 children aged 11–15 years. Importantly, Aartun et al. (2016) examined this association both cross-sectionally and longitudinally, measured site-specific pain, and included a bigger sample size than ours, which all reduce the risk of bias relative to our study and may explain the difference in conclusions. However, the difference in age of participants and focus on spinal pain by Aartun et al. (2016) may also contribute to differences in findings.

Two prior studies investigated the association between subjective PA intensity and pain in children, and, similar to our findings, Silva et al. (2017) reported that more time spent in MPA was significantly associated with a higher probability of reporting neck, shoulder, low back, wrist, hip, knee and ankle/foot pain. Findings from the present investigation also corroborate other results from Silva et al. (2017) showing that more time spent in VPA was significantly associated with a higher probability of reporting pain on shoulders, mid back, knees and ankles/feet. The findings may be similar between studies because a similar design was used. In the study by Silva et al. (2017), children were asked to rate their pain over the past seven days and at the same time were asked if they participated in moderate and/or vigorous physical activities. The use of an objective measure of PA in our study strengthens these findings. However, any association between PA and pain may be incidental given the cross-sectional designs used in both studies. Indeed, Swain et al. (2016) found that reduced participation in self-reported MVPA was associated with the presence of back pain, headache and stomach-ache in adolescent girls and combined headache and stomach-ache or headache in adolescent boys. Unlike our study and the study by Silva et al. (2017), Swain et al. (2016) defined the presence of pain as experiencing pain at least every month over six months. Physical activity was also self-reported, and adolescents were dichotomized as active or underactive (Swain et al., 2016), which may have resulted in misclassification of PA. These factors may explain the difference in findings between studies and also highlight the potential impact of the methods used to measure pain and PA on findings.

Similarly to our findings, Nauta et al. (2017) found that objectively measured MVPA did not predict acute upper extremity injury risk. However, their study did not examine separate associations between MPA, VPA and injury. Indeed, a recent systematic review and meta‐analysis (Farooq et al., 2020) found that most studies did not investigate these MPA and VPA associations separately. Lowry et al. (2007) found that high frequency participation in self-reported MPA was associated with decreased odds of injury among boys, and medium and high frequency self-reported VPA were associated with increased odds of injury among girls. This suggests that it is important to examine the separate associations between MPA, VPA and injury. While we hypothesised that objectively measured time in VPA would be more associated with injury than MPA, because of increased joint loading during participation in VPA, our findings did not support this hypothesis.

No prior study assessed if the association between PA and pain or the association between PA and injuries differs according to children’s weight status. The literature shows that OW and OB are related to musculoskeletal pain in children (Stovitz et al., 2008). Although we hypothesised that PA would be more likely to be associated with pain among children with OW/OB than those with normal weight as a result of increased joint loading due to excess weight (Lerner et al., 2016), our results did not support this hypothesis even after separately considering the different intensities of PA. It is possible that type of PA (unexamined in our research) is more important to children’s pain/injury associations than PA intensity. It is also possible that an association between PA, pain and injuries exists among children with OB but not for children who are OW. Our sample included only five children with OB and thus we analyzed children with OW/OB collectively. Further studies are needed to examine if PA type and intensity is associated with pain and injuries among children with obesity. Further, there was considerable variation in time spent in PA among children with OW/OB, and this variation was greater among these children than among children with normal weight. This factor in combination with the smaller number of children with OW/OB in our study relative to the number with normal weight, may explain why we found an association between MPA and pain in children with normal weight but not among children with OW/OB.

## Limitations and Directions for Further Research

Limitations of this study include the aforementioned omission of types of PA, the small number of children with OW/OB that prevented their separate analyses, and its cross-sectional design. The design prevents us from making causal determinations in the relationship between PA and any pain/injuries that may develop in the future. Also, we measured pain over seven days, making it harder to determine whether there may have been associations between PA and acute or temporary pain during or immediately after PA. Additionally, children were asked to recall injuries over the past 12 months, which may have been difficult for them to recall, resulting in inaccurate estimates of the number of past injuries experienced. The association between PA, pain and injuries should be examined in future studies by measuring PA and pain/injuries at multiple, non-coincident, timepoints. The low response rate and the lack of information with regards to pain location are also limitations of the present study. Lastly, our participant sample was relatively small and recruited from one geographical area. However, mean time in VPA in the present study (17.1 min) is similar to that observed in a recent study of 425 children aged 9–11 years in England (20.9 min) (H. J. Wilkie et al., 2018). Also, the prevalence of OW/OB in our sample at 35% was identical to the prevalence of OW/OB in children aged 10–11 years in 2018/19 (35.2%) reported by the National Child Measurement Programme in England (Lifestyle Statistics Team, 2019). Future researchers should review these limitations to address them specifically in subsequent studies.

## Conclusions

Findings from this study suggest that objectively measured VPA is associated with pain among children aged 8 to 12 years. MPA may also be associated with pain in children with normal weight. We did not find evidence that MPA was associated with pain in those with OW/OB, though this may be related to a small sample of children with OW/OB. We also found no evidence that objectively measured MPA or VPA are associated with injury in children. These findings do not indicate any causal relationship between PA and pain, and there is a need for new research to address specified limitations in this study. While these findings should be replicated in longitudinal studies, they suggest that pain resulting from children’s participation in VPA warrants consideration when developing PA interventions for children.
